# Supplementary material for: Quantum-mechanical exploration of the phase diagram of water
Source: Nat Commun. 2021 Jan 26;12:588. doi: 10.1038/s41467-020-20821-w (PMC7838264; doi:10.1038/s41467-020-20821-w)
Supplement: Supplementary file 2 — Description of Additional Supplementary Files [file 41467_2020_20821_MOESM2_ESM.pdf]

Description of Additional Supplementary Files

Supplementary Data 1

DFT input files, example ice configurations studied, a Mathematica notebook used to collate the results, and numerical results of the free-energy computations.
